# Radiation therapy and secondary malignancy in Li‐Fraumeni syndrome: A hereditary cancer registry study

**DOI:** 10.1002/cam4.3427

**Published:** 2020-09-15

**Authors:** Peter G. Hendrickson, Yukun Luo, Wendy Kohlmann, Josh Schiffman, Luke Maese, Andrew J. Bishop, Shane Lloyd, Kristine E. Kokeny, Ying J. Hitchcock, Matthew M. Poppe, David K. Gaffney, Randa Tao

**Affiliations:** ^1^ Department of Radiation Oncology University of Utah‐ Huntsman Cancer Institute Salt Lake City UT USA; ^2^ Department of Pediatric Hematology and Oncology University of Utah‐ Huntsman Cancer Institute Salt Lake City UT USA; ^3^ Department of Radiation Oncology University of Texas MD Anderson Cancer Center Houston TX USA

**Keywords:** LFS, Li‐Fraumeni syndrome, p53, radiation, RT‐induced malignancy

## Abstract

**Background:**

Li‐Fraumeni Syndrome (LFS) is a rare cancer‐predisposing condition caused by germline mutations in *TP53*. Conventional wisdom and prior work has implied an increased risk of secondary malignancy in LFS patients treated with radiation therapy (RT); however, this risk is not well‐characterized. Here we describe the risk of subsequent malignancy and cancer‐related death in LFS patients after undergoing RT for a first or second primary cancer.

**Methods:**

We reviewed a multi‐institutional hereditary cancer registry of patients with germline *TP53* mutations who were treated from 2004 to 2017. We assessed the rate of subsequent malignancy and death in the patients who received RT (RT group) as part of their cancer treatment compared to those who did not (non‐RT group).

**Results:**

Forty patients with LFS were identified and 14 received RT with curative intent as part of their cancer treatment. The median time to follow‐up after RT was 4.5 years. Fifty percent (7/14) of patients in the curative‐intent group developed a subsequent malignancy (median time 3.5 years) compared to 46% of patients in the non‐RT group (median time 5.0 years). Four of seven subsequent malignancies occurred within a prior radiation field and all shared histology with the primary cancer suggesting recurrence rather than new malignancy.

**Conclusion:**

We found that four of14 patients treated with RT developed in‐field malignancies. All had the same histology as the primary suggesting local recurrences rather than RT‐induced malignancies. We recommend that RT should be considered as part of the treatment algorithm when clinically indicated and after multidisciplinary discussion.

## Graphical abstract

Forty patients with LFS were identified and 14 received RT with curative intent as part of their cancer treatment. We found that four of 14 patients treated with RT developed in‐field malignancies. All had the same histology as the primary suggesting local recurrences rather than RT‐induced malignancies.

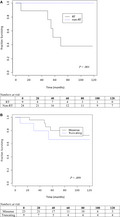

## 1 INTRODUCTION

Li‐Fraumeni syndrome (LFS) is a rare autosomal‐dominant condition caused by inherited mutations in *TP53,* or less commonly, CHEK2.^1^, ^2^ The wild‐type protein product of *TP53*, called p53, is a potent tumor‐suppressor that mediates cell death in response to DNA damage. In this capacity, p53 is regarded as the “guardian of the genome,” and when mutated, the cell becomes vulnerable to DNA damage and subsequent cancer development.

To date, more than 250 distinct LFS‐causing mutations have been described in the literature including many different mutational types (nonsense, frameshift, splice‐site, large deletions, etc) throughout all four protein domains of p53.^3^, ^4^, ^5^ The most common inherited mutations are of the missense variety and occur in exons 5‐8, corresponding to the DNA binding domain of the protein.^6^ These mutations perturb the normal protein folding pattern, resulting in ineffective DNA binding and transcriptional activation. While most mutant p53 proteins display a dominant‐negative effect on wild‐type protein through the formation of hetero‐tetramers, some mutants can promote malignant cellular transformation by upregulating prooncogenic gene expression.^7^

Li‐Fraumeni syndrome is highly penetrant with sequelae more pronounced in women than in men due to the high incidence of breast cancer.^8^ Although the spectrum of cancers is wide, malignancies of the breast, brain, adrenal glands, blood (leukemias), bones (osteosarcomas), and soft‐tissues (soft tissue sarcomas) are the most common and are classically associated with the disease.^8^ The risk of developing a primary cancer is 50% by age 31 in females and age 46 in males.^9^ Li‐Fraumeni syndrome patients, however, also have a heightened risk of developing secondary and even tertiary cancers throughout their lifetime.^10^ Intuitively, this risk is thought to increase with exposure to DNA‐damaging agents such as ionizing radiation and chemotherapy.^11^

Although radiation‐induced cancer is a rare event in the normal population, accounting for less than 5% of all treatment‐related secondary malignancies, it is suspected to be much more common in populations with cancer predisposition syndromes such as LFS.^12^ One of the largest retrospective studies by Bougaurd et al of 64 French LFS patients reported the incidence of secondary tumors in a previous radiation field at 30%, with a mean time to development of 10.7 years.^13^ This risk is supported by work done in preclinical models which show that *Trp53* heterozygous mice are more susceptible to radiation‐induced tumors.^14^, ^15^Whereas almost half of all adult cancer patients nationwide receive radiotherapy (RT) as an integral part of their cancer treatment, RT utilization has historically been more selective in LFS patients due to concerns for second malignancies. Few well‐controlled studies exist looking directly at outcomes in patients with inherited *TP53* mutations after RT, considering the limited use of treatment and the relatively low frequency of disease. As a result, there are no specific evidence‐based RT treatment guidelines for the management of LFS patients with cancer. Here we explore the effects of RT in LFS patients with regards to the development of subsequent malignancies and survival.

## 2 METHODS

We reviewed a cohort of patients with germline *TP53* mutations within a multi‐institutional hereditary cancer registry hosted at our institution. We obtained outside records on patients treated at other institutions. Institutional review board approval was obtained for this study. We assessed the rate of subsequent malignancy and death in patients who received RT (RT group) as part of their cancer treatment against those who did not (non‐RT group). The term “subsequent malignancy” was used to describe all cancer diagnoses after the first primary cancer irrespective of its temporal or histological relationship to the first. Time to follow‐up, time to subsequent malignancy, and time to death measurements were calculated from the date of the first cancer diagnosis. The modified Cahan's criteria described by Singh et al were considered to determine whether or not a “subsequent malignancy” was radiation induced.^16^ Briefly, subsequent cancers needed to be of a different histology and must have occurred in a previously irradiated field after a sufficient latency period. As the latency period for radiation‐induced malignancy in LFS patients is not clear, we did not consider this criterion when categorizing subsequent malignancies. Subsequent tumors with the same histology as the primary tumor were determined to be “recurrent cancers” if they occurred locally or “metastatic cancers” if they occurred distantly. We further describe whether subsequent tumors occurred within or outside the radiation treatment fields. Subsequent tumors that were histologically distinct from the primary tumor and occurred outside of the radiation field were called “new primary cancers.” Prognostic groups were determined retrospectively based on all available cancer data including cancer size, location, grade, and stage as well as patient demographics. This categorized patients into preinvasive, favorable and unfavorable prognostic groups with 5‐year overall survival (OS) for the favorable group in a range of 80%‐98% whereas 5‐year OS for the unfavorable group was 30%‐65%. All patients were confirmed to have a pathogenic or likely pathogenic variant in *TP53* from a commercial genetic testing laboratory.

## 3 RESULTS

### 3.1 Patient characteristics

Our cohort included 40 LFS patients, including 14 males and 26 females from 27 different families. A total of 21 distinct *TP53* mutations were present in our study. Twenty‐eight (70%) patients had missense mutations in the DNA binding domain or oligomerization domain of p53 and 11 (28%) had truncating mutations caused by nonsense, frameshift, splice site, or large deletion mutations (Table 1).

**Table 1:** Patient characteristics

	Non‐RT patients n = 24	RT patients n = 16	All Patients n = 40
Gender			
Male	5	9	14
Female	19	7	26
Age at first cancer (year)			
Mean	21.5	23.7	22.4
Median	16.5	22.5	20.5
Range	0‐51	1‐47	0‐51
First cancer type			
Breast	8 (33.3%)	3 (18.8%)	11 (27.5%)
Brain/CNS	4 (16.7%)	3 (18.8%)	7 (17.5%)
STS	1 (4.2%)	5 (31.3%)	6 (15.0%)
Bone	3 (12.5%)	2 (12.5%)	5 (12.5%)
Adrenocortical	5 (20.8%)	1 (6.3%)	6 (15.0%)
Blood	1 (4.2%)	0 (0.0%)	1 (2.5%)
Other	2‐ [1] RCC, [1]NSCLC(8.3%)	2‐CRC (12.5%)	4 (10.0%)
TP53 mutation			
Missense			
DNA binding Domain	17	8	25 (62.5%)
Tetramerization Domain	1	2	3 (7.5%)
Nonsense	2	1	3 (7.5%)
Frameshift	0	1	1 (2.5%)
Large deletion	3 (exon 1)	2 (exon 1)	5 (12.5%)
Splice	1	1	2 (5.0%)
Unknown	0	1	1 (2.5%)

Abbreviations: CRC, colorectal cancer; NSCLC, nonsmall cell lung cancer; RCC, renal cell carcinoma; RT, radiation therapy.

The median age of a first cancer diagnosis was 20.5 years. Breast cancer was the most common first cancer diagnosis at 28%. The next most common diagnoses were CNS/brain cancer (18%), soft tissue sarcoma (15%), adrenal cortical carcinoma (15%), and bone tumors (13%) (Table 1). A total of 23 patients (58%) developed a subsequent malignancy, which included new primary cancer(s) (n = 15, 65%) or recurrent/metastatic disease (n = 8, 35%). Fifteen patients (38%) developed a second primary cancer (median age 35 years) and five (13%) developed a third primary cancer (median age 44 years).

Sixteen patients (40%) received RT as part of their cancer treatment; 14 with curative intent (including definitive and adjuvant therapy) and two patients with advanced or metastatic disease were treated with palliative intent (Table 2). The median time to follow‐up after RT was 4.5 years. Of those treated with curative intent, nine patients received RT for a first primary cancer, two for a second primary cancer, and five were treated for recurrent or metastatic disease. Among the patients who were treated with recurrent disease, four had local recurrences after surgery alone: one patient had a grade II astrocytoma, one patient with a stage I breast cancer, one patient with a recurrent glioma treated with a subtotal resection, and one patient with an osteosarcoma who had a positive margin after surgery. One patient (Table S1: patient 14) was treated twice‐ once for a recurrent brain tumor and then again for a third primary malignancy. The median dose among all patients was 50.4 Gy (range 15‐66 Gy) and all but one patient was treated with standard fractionation with 1.8‐2 Gy per fraction. Radiation techniques used included 3D conformal, intensity modulated radiation therapy (IMRT), and one patient underwent intraoperative RT with a HAM applicator. All non‐RT patients were treated with primary tumor resection/ablation or in the case of one patient with myelodysplastic syndrome, a stem cell transplant. Six (25%) patients also received chemotherapy, of whom three (50%) developed subsequent malignancies compared to eight (50%) treated with resection/ablation alone. The only difference between the RT and non‐RT groups was the frequency of unfavorable cancer prognoses (*P* < .001) which was determined retrospectively by a cohort of oncologists (Table 3). Age, sex, and mutation type, were not associated with receipt of RT (*P *> .05).

**Table 2:** Application of radiation therapy (RT)

	RT patients n = 16	Patient numbers (from Supp Table S1)
Intent		
Curative	14	1‐14
Palliative	2	15‐16
Cancer treated		
First primary	9	1‐9
Second primary	2	10‐11
Recurrent/metastatic disease	5	12‐16
Time to follow‐up after RT (mo)		
Mean	58.4 (4.9 years)	
Median	54.5 (4.5 years)	
Range	2‐144	
Subsequent malignancy after RT (# of patients)		
Recurrent disease (same/similar histology)		
Local (in‐field)	5	2,4,5,12,14
Distant/Metastasis	1	5
New primary (different histology)		
Local (in‐field)	0	
Distant/out‐of‐field	3	3,7,14

Abbreviations: RT, radiation therapy.

**Table 3:** Prognostic groups by histology and stage

Preinvasive	Favorable (5‐year overall survival 80%‐98%)	Unfavorable (5‐year overall survival 30%‐60%)
		
DCIS	Invasive ductal carcinoma (stage I‐III)	Lung adenocarcinoma (unknown)
Colon intra‐mucosal adenocarcinoma	Phyllodes sarcoma (stage III)	Colon adenocarcinoma (stage IIIC)
Myelodysplastic syndrome	Renal Cell Carcinoma (Stage I)	Astrocytoma (grade 3)
	Astrocytoma (grade 2)	Choroid plexus carcinoma (grade 3)
	Dysembryoplastic neuroepithelial tumor (low‐grade)	Osteosarcoma of right tibia (unknown)
	Pleomorphic xanthoastrocytoma (grade 2)	Osteosarcoma of left tibia (high‐grade, stage IIa)
	Chordoma	Osteosarcoma of rib (moderate‐grade, stage IIb)
	Giant cell tumor of left femur (unknown)	Embryonal rhabdomyosarcoma of pelvis (stage IV)
	Adrenocortical carcinoma	Pleomorphic liposarcoma of foot (high‐grade, stage II)
	Rhabdomyosarcoma of orbit	Pleomorphic sarcoma of pelvis (high‐grade, stage III)
	Embryonal rhabdomyosarcoma of scapula (stage II)	Pleomorphic sarcoma of retroperitoneum (high‐grade, stage III)

### 3.2 Subsequent malignancies and survival outcomes

Among the RT group, seven patients (S1: patients 2,3,4,5,7,12, 14) developed a subsequent malignancy after curative‐intent RT resulting in a cumulative incidence of 50%. Of these, five occurred in the radiation treatment field and were also of the same histology as the primary tumor; four sarcomas and one astrocytoma (S1: patients 2,4,5,12,14). Based on the provided clinical scenarios, these were consistent with recurrent disease. The remaining three patients with subsequent malignancies developed new primary cancers of which all occurred outside the radiation field including two sarcomas and a B‐cell acute lymphoblastic leukemia (B‐ALL) that occurred after chemotherapy and radiation for a primary breast cancer (S1: patients 3,7,14). Interestingly, the anthracycline‐based chemotherapy received by this patient, but not breast RT, has been associated with a significantly increased risk of bone marrow neoplasms.^17^ Therefore, none of the malignancies occurring in patients who received RT were classified as RT associated. In order to assess outcomes after RT, the patients who received RT for their first primary cancer (S1: patients 1‐9) were evaluated and compared to patients who did not receive RT calculated from the time of diagnosis (Table 4). Five (56%) patients in the RT group who received treatment for their primary cancer (S1: 2,3,4,5,7) compared to 11 (46%) patients in the non‐RT group developed a subsequent malignancy (*P *= .71).

**Table 4:** Outcomes after treatment with curative‐intent radiation therapy (treated for first primary cancer)

	Non‐RT n = 24	RT n = 9	*P*‐value
Subsequent malignancy (# of pts)			
Recurrent disease (same histology)			
Local	1	3	
Distant/Metastasis	0	0	
New primary (different histology)			
Local	0	0	
Distant	10	2	
Total	11 (45.8%)	5 (55.5%)	.7080
Time to subsequent malignancy (months)			
Mean	103.6 (8.6y)	42.6 (3.5y)	
Median	60 (5.0y)	39 (3.3)	
Range	11‐329	17‐90	
Number of deaths to date	1	5	**<.001**
Time to death from first primary (months)			
Mean	347 (28.9y)	47.4 (3.9y)	
Median	347 (28.9y)	54 (4.5y)	
Range	na	8‐57	
Prognosis at diagnosis			
Pre‐invasive	4 (16.7%)	0 (0.0%)	
Favorable	17 (70.8%)	3 (33.3%)	
Unfavorable	3 (12.5%)	6 (66.7%)	**.01**
10‐year overall survival	100%	44.4%	**<.001**

Bold values indicates *P* ‐values < .05.

Abbreviations: pts, patients; RT, radiation therapy; y, years.

**Figure 1:**
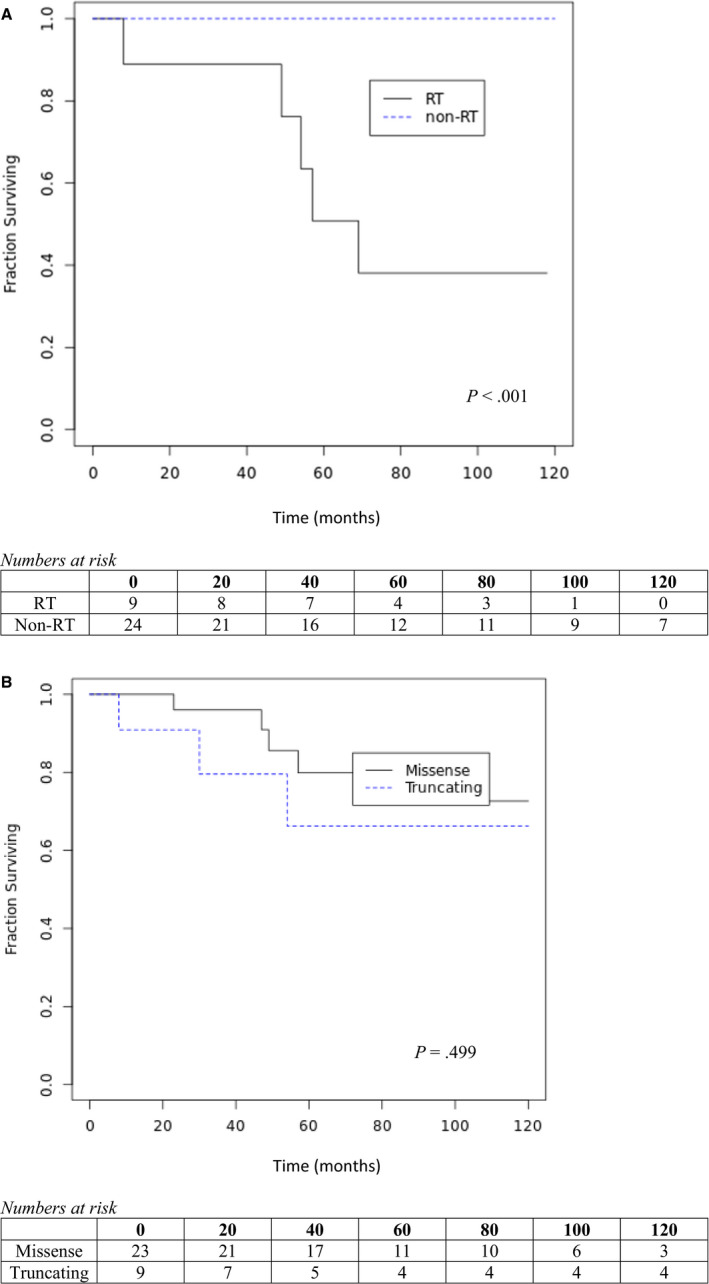
A, Ten‐year overall survival after first primary cancer diagnosis. Abbreviations: RT, radiation therapy; non‐RT, nonradiation therapy. B, Ten‐year overall survival after first primary cancer diagnosis by mutation status

Regarding survival, while only one death has occurred to date in the non‐RT group (28.9 years after the patient's first cancer diagnosis), five deaths have occurred in the RT group (S1: 3,4,5,7, 9). All five deaths in this group were due to cancer progression with a median time to death of 4.5 years. Accordingly, the 10‐year overall survival after first cancer diagnosis for LFS patients who did not receive RT was 100% compared to 44% in the patients treated with RT (*P* < .001, Figure 1A). This observation is likely explained by the aggressive nature of disease in patients who were selected to receive RT. Furthermore, among all patients, analysis based on mutation type demonstrated no statistically significant differences in survival between missense versus truncating mutation carriers (*P* = .499, Figure 1B).

Despite poorer survival outcomes among the RT group, there was no statistically significant difference between the RT and non‐RT group with regards to the development of a subsequent malignancy (OR 1.48, 95% CI = 0.32‐6.90, *P* = .62). This result held true regardless of the mutation type: missense (OR 2.36, 95% CI = 0.31‐17.9, *P *= .41) or truncation (OR 0.25, 95% CI = 0.01‐4.7, *P *= .36). There was, however, a significant difference in the cancer prognosis between the two groups. Whereas the majority of patients (67%) in the RT group had cancers with “unfavorable” prognoses, the same was true for only a minority of patients (13%) in the non‐RT group (*P* < .001).

## 4 DISCUSSION

Radiation therapy remains a mainstay of cancer treatment alongside surgical resection and chemotherapy often as an adjuvant to prevent locoregional recurrence.^18^ In patients with LFS who have a high predisposition for cancer development, RT has historically been omitted out of concern for inducing a secondary malignancy. Despite a strong theoretical rationale, however, there may be inadequate clinical data to support this concern and practice. Based on our series, which is albeit limited by a short follow‐up period discussed more below, we observed no RT‐associated malignancies. We also observed two patients with local recurrences after incomplete surgery that may have been prevented with adjuvant RT. Therefore, with critical multidisciplinary input and proper patient counseling, we argue that RT should at least be considered for patients with LFS in the setting of an aggressive cancer and when an equally efficacious alternative treatment is not available. For example, in the setting of breast cancer, postmastectomy radiation (PMRT) is recommended for patients with inflammatory breast cancer or when there is involvement of four or more lymph nodes, and it is strongly considered for patients with fewer involved nodes, large primary tumor (>5 cm), or positive margins that cannot be cleared by reexcision. This is due to the high incidence of disease recurrence in these populations which is difficult to treat and tied to poor outcomes because of distant disease spread. The current guidelines according to the American Society of Radiation Oncology, however, are to withhold PMRT in LFS patients based on the assumption that the risk of radiation‐associated malignancy is greater than the risk of recurrent disease.^19^ In our study, although underpowered for statistical analysis, the risk of developing a local recurrence after mastectomy alone (22% at median follow‐up time of 29 months) appears to be greater than the risk of a radiation‐associated malignancy (0% at median follow‐up time of 87 months). In patients without germline p53 mutations, the benefit of PMRT appears to be greatest for those with four or more positive lymph nodes where it has been shown to reduce locoregional recurrence by 19% and breast cancer mortality by 9%.^20^ Future retrospective studies might examine which LFS patients with breast cancer are at greatest risk of locoregional recurrence in order to elucidate who would most benefit from PMRT and in whom it should be omitted.

In this work, we observed a rate of subsequent malignancy of 50% in LFS patients receiving RT. However, four of seven patients had a local recurrence of their primary disease and three of seven patients had a subsequent malignancy outside the RT field. This distinction is important because previous studies did not clearly define their reported subsequent/secondary malignancies. For example, in a large LFS population study, Bougeard et al reported that 19 of 64 (30%) LFS patients who received RT developed a subsequent malignancy within a prior radiation field.^13^ Though alarming, it is unclear from this study what fraction of these cancers represent true radiation‐associated secondary malignancies versus recurrent or progressive disease. This same limitation applies to a review by Suri et al which reported a cumulative incidence of secondary malignancy after RT as high as 48% (which is similar to our subsequent malignancy rate prior to further classification) in 11 out of a total of 23 pooled patients from 10 articles and case reports; however, they did not define what constituted a secondary malignancy.^21^ Despite the limitations in these studies, it is important to note that the numbers are consistent with a smaller cohort studies such as one by Heymann et al that reported specifically on the incidence of radiation‐induced cancers in LFS patients treated for primary breast cancer.^22^ In that study, two of six (33%) LFS patients developed new primary tumors in the radiation field, both of which were temporally and histologically compatible with radiation‐induced malignancies.

In this study, four of 14 (29%) LFS patients who received curative RT developed a subsequent cancer in the radiation field. None of these “in‐field” cancers, however, met criteria for or were consistent with a radiation‐induced malignancy. Of the subsequent cancers, all were histologically identical to the primary tumor. These results go against conventional wisdom and previously published preclinical and clinical reports. One explanation for the discrepancy is that prior studies did not differentiate between new primary cancers and recurrent disease as discussed above. As RT has historically been reserved for LFS patients with more aggressive disease, it is reasonable to posit that previous numbers were skewed by primary disease recurrence or progression which would be expectedly higher in the RT‐treated population. A second explanation is that the median follow‐up time of 4.5 years in this study may be too short to capture radiation‐associated secondary malignancy. In a population without documented germline p53 mutations, the latency period for a radiation‐induced malignancy is estimated to be between 10 and 60 years.^18^ Although the latency period is likely shorter in the LFS population based on observations in *Tp53* heterozygous mice, it is plausible that a longer follow‐up time in this study could lead to a higher observed rate of radiation‐associated malignancies. This is supported in a study by Bougeard et al in which the average in‐field subsequent cancer occurred 10.7 years after RT.^13^ A third consideration is how differences in patient demographics (i.e. age at exposure, sex, etc) as well as radiation site, dose, and regimen influence the rate of secondary malignancy in LFS patients. The incidence of radiation‐induced sarcoma in patients without germline p53 mutations tends to increase with decreasing age at treatment, use of concurrent chemotherapy, and increasing radiation dose.^23^

Life expectancy in LFS is highly variable, dependent on the type of cancer, and age at diagnosis. Prior work has suggested that this phenotypic heterogeneity can be linked to the type of *TP53* mutation and unequal gain of function effects.^24^ Here we find no significant difference in life expectancy based on the *TP53* mutation type. Few studies have directly compared survival outcomes in LFS patients with respect to RT. Presumably, this is because of the inherent selection bias as RT is typically reserved for LFS patients with more aggressive cancers. This bias was highlighted in a study by Bahar et al which reported a significant survival disadvantage associated with the use of RT in LFS patients with Choroid plexus carcinoma (CPC).^25^ Although cancer‐related mortality of 82% in the RT group compared to 41% in the non‐RT group was observed, 44% of the deceased patients in the RT group were treated for recurrent disease and thus likely had poor prognoses irrespective of treatment. In this study, we looked at survival outcomes among patients who received RT for a first primary cancer against those who never received RT. To date, already five deaths have occurred in the RT group (56%, median 4.2 years after first cancer diagnosis) while only one death has occurred in the non‐RT group (4.1%, 28.9 years after first cancer diagnoses). A common explanation or assumption made in the literature is that RT exposure increases the risk of subsequent malignancy in the LFS population to adversely affect survival. This explanation, however, is not supported by our data as we found no statistically significant difference in the number of subsequent cancers between the RT and non‐RT groups (67% versus 46%, *P* = .44). Instead, this difference was influenced by the different prognoses of cancers treated in the RT versus non‐RT group. We also found that the patients who received RT had worse cancer prognoses. Thus, it is essential to note this source of selection bias in future studies including LFS patients treated with RT.

Our study contributes to the literature on LFS patients treated with RT given the comparatively large cohort and detailed descriptions of subsequent malignancies. However, it has several limitations inherent to all retrospective studies. This study included a heterogeneous patient population with several different types and stages of primary and subsequent malignancies, though this is reflective of the “real‐world” complexities of the LFS patient population. Additionally, a limitation of this study is that it was not possible to unequivocally distinguish between disease recurrences versus radiation‐induced cancer that was of the same histology as that of the primary. However, this is often the case in any study and we included the clinical information on why subsequent malignancies were more consistent with recurrent disease rather than RT‐induced, which is often not described in the literature. In future work, the use of DNA sequencing and mutational signatures may be employed to reliably distinguish recurrent disease from radiation‐induced cancers.^26^, ^27^, ^28^ Another potential limitation is the limited follow‐up time. Although a median follow‐up time of 4.5 years after completion of RT is comparable to other series, it still may not be adequate to capture the full latency period for the development of radiation‐associated malignancies. Thus, we have plans for a future study to update the outcomes of the remaining members of this cohort after a longer follow‐up period, ideally with a median follow‐up significantly longer than 4 years based on the modified Cahan's criteria (Singh et al 2017). A strength of this study is the inclusion of the details of the subsequent malignancies observed instead of only reporting whether or not they occurred in the radiation field. The number of patients included in our study, 40 total and 16 treated with RT, is quite large when compared to historical studies as these primarily consist of series limited to fewer than five patients or one patient case reports.^29^, ^30^, ^31^ Thus, another strength of the study is that we present data from a relatively large cohort of LFS patients.

## 5 CONCLUSIONS

We found that the rate of a subsequent malignancy was not significantly different between LFS patients who received RT compared to those who did not. In fact, none of the subsequent malignancies in patients receiving RT could be categorized as an RT‐associated malignancy; all the subsequent malignancies in the radiation fields were consistent with disease recurrence. Patients treated with RT did have significantly worse OS outcomes despite not having a higher rate of developing a subsequent malignancy, which is likely a consequence of patients with a worse prognosis being selected to receive RT. Future studies with longer follow‐up times and larger sample sizes enabling more control over disease prognosis are needed before changes to the current LFS treatment recommendations can be suggested. Additionally, future studies could utilize advances in genotyping to help distinguish recurrent disease from a radiation‐induced secondary malignancy. At this point, our data provide preliminary evidence to suggest RT should not be withheld in patients with LFS when clinically indicated.

## ETHICS APPROVAL

Institutional review board approval was obtained prior to commencing this study.

## CONFLICTS OF INTEREST

Josh Schiffman is the cofounder, shareholder, and employed by PEEL Therapeutics, Inc (biotech company), and cofounder and shareholder of *ItRunsInMyFamily.com* (online family history tool). Matthew Poppe is an investor in PEEL Therapeutics, Inc (biotech company) and has stock ownership in Inovio, Merck, Moderna, Sanofi, and Astrazeneca. Randa Tao has served on a cholangiocarcinoma advisory board and received consulting fees from QED Therapeutics and The Lynx Group, outside this work.

## AUTHOR CONTRIBUTIONS

All authors contributed to the interpretation of the data, reviewed and revised the manuscript, and give final approval to the version to be published.

## Supporting information

Table S1

## Data Availability

The data that support the findings of this study are available on request from the corresponding author. The data are not publicly available due to privacy or ethical restrictions.
