# Supplementary material for: Radiation therapy and secondary malignancy in Li‐Fraumeni syndrome: A hereditary cancer registry study
Source: Cancer Med. 2020 Sep 15;9(21):7954–63. doi: 10.1002/cam4.3427 (PMC7643676; doi:10.1002/cam4.3427)
Supplement: Supplementary file 1 — Table S1 [file CAM4-9-7954-s001.docx]

**Supplementary Table 1- Clinical Scenarios of Patients Treated with Radiation Therapy (RT)**

Patient 1

A 40-year-old female with an extensive family history of cancer on the paternal side was diagnosed with stage IIB invasive ductal carcinoma of the left breast and DCIS of the right breast in 2010. She was treated with bilateral mastectomy, chemotherapy, and radiation receiving 50 Gy in 25 fractions to the left chest wall and regional nodes. In 2011, while undergoing radiation treatment planning, a thyroid mass was incidentally discovered prior to the start of RT. Biopsy results showed papillary thyroid carcinoma and she was treated with total thyroidectomy shortly after she completed her RT course. In light of her family and personal cancer history, she was tested for LFS in 2013 which revealed a 323_329dupGTTTCCG mutation in the TP53 gene. At last follow up in 2014, the patient was doing well with no evidence of disease or subsequent malignancies.

Patient 2

A 19-year-old female with a distant family history of cancer was diagnosed with a Stage III (T2bN0M0) high-grade pleomorphic sarcoma of the left retroperitoneum in 2010. She initially received preoperative radiation with 50.4 Gy in 28 fractions that was completed in December 2010 and the sarcoma was then resected in January in 2011 with negative margins. In 2013 she was discovered to have a 1040C>A mutation in the TP53 gene. She remained disease free until 2018 when a new left-sided retroperitoneal mass was discovered on screening MRI suspicious of recurrent disease. Biopsy showed recurrent/residual high-grade pleomorphic sarcoma. This was initially treated with cryoablation in May 2018 but she had local progression of disease. She underwent surgical resection in October 2018 and was started on chemotherapy. Her disease continued to progress and she was started on a second course of radiation therapy but only completed about half of the planned treatments in June 2019 in order to undergo surgery for persistent pain. As of last follow up in July 2019, the patient was alive but with advanced disease.

Patient 3

A 46-year-old male was diagnosed with a stage II, high-grade pleomorphic liposarcoma of his right foot in 2008 which led to the discovery of LFS. The tumor was surgically resected and patient underwent intraoperative RT with a HAM applicator receiving 15 Gy in 1 fraction. In 2010, he developed a cutaneous leiomyosarcoma on his right back that was excised and then in 2013, he was discovered to have an esophageal adenocarcinoma with metastases to the liver. He passed away 1 year later in 2014.

Patient 4

A 46-year-old female with LFS due to a 751A>C mutation in the *TP53* gene was diagnosed with a stage III high-grade pleomorphic sarcoma of the ischiorectal fossa in 2010. She was treated with surgical resection as well as pre- and post-op RT receiving 50.4 Gy in 28 fractions and 16 Gy in 8 fractions, completing treatment in 2011. In 2013, a screening MRI demonstrated a new right ischiorectal fossa mass with biopsy showing a high-grade pleomorphic sarcoma. She was treated palliatively and passed away in November 2014.

Patient 5

A 4-year-old female with no family history of cancer was diagnosed with a stage IV high-grade embryonal rhabdomyosarcoma of the pelvis with lung metastasis in 2013. She was treated with chemotherapy and received concurrent whole pelvis and whole lung RT to 36 Gy in 20 fractions and 15 Gy in 10 fractions, respectively. In 2016, she received a diagnosis of LFS caused by a splice site mutation (673-1G>T) in the *TP53* gene. A screening MRI in June 2016 showed interval growth of her pelvic mass with new metastases to the pelvic bone, femur, and ilio-psoas muscle. She was treated palliatively and passed away in September 2017.

Patient 6

A 33-year-old male with an extensive family history of cancer and personal history of basal cell carcinoma was diagnosed with an anaplastic astrocytoma in 2009. He underwent surgical resection and then received adjuvant chemoradiation therapy at an outside hospital system. Shortly thereafter he received an LFS diagnosis with the discovery of a familial 584T>C mutation in the *TP53* gene. At last follow-up in 2019, he is doing well with no evidence of disease or subsequent malignancies.

Patient 7

A 47-year-old female without a notable family history of cancer was diagnosed with a stage II invasive ducal carcinoma of the left breast in 2011. She was treated with lumpectomy, chemotherapy (receiving taxotere, carboplatin, and herceptin), and radiation therapy with 50 Gy in 25 fractions to the left breast. In January of 2016, she was diagnosed with LFS caused by 824G>A mutation in the *TP53* gene and in March was found to have a B-cell lymphoblastic leukemia. She was treated with chemotherapy but passed away from complications in August of 2017.

Patient 8

A 2-year-old male was diagnosed with orbital rhabdomyosarcoma in 2010 treated at an outside hospital with resection, chemotherapy, and proton beam RT. In 2016, the patient was diagnosed with LFS caused by a 743G>A mutation in the *TP53* gene. At last follow up in November 2017, he had no evidence of disease or subsequent malignancies.

Patient 9

A 1-year-old male with an extensive family history of cancer was diagnosed with a WHO grade III choroid plexus carcinoma in May 2011 leading to the discovery of LFS caused by a 702C>G mutation in the *TP53* gene. He was treated with surgical resection and chemoradiation therapy receiving 50.4 Gy in 28 fractions. Despite aggressive treatment, he passed away in February 2012 from complications of his cancer.

Patient 10

A 19-year-old male with known LFS caused by a 751A>C mutation was found to have a colonic intramucosal adenocarcinoma in 2008 that was treated with polypectomy. Three years later in December 2011, he was diagnosed with a glioblastoma that was treated with surgical resection and chemoradiation therapy to 59.4 Gy in 33 fractions, completing treatment in July 2012. Despite treatment, his disease rapidly progressed and he passed away in October of 2012.

Patient 11

A 2-year-old female was diagnosed with adrenocortical carcinoma in 1996 treated with adrenalectomy. At the age of 15, in 2010, she was found to have a stage IIIA right medullary breast cancer which led to the diagnosis of her LFS caused by a 818G>A mutation. This was treated with mastectomy, chemotherapy, and radiation therapy. She received 48 Gy in 24 fractions to the chest wall and at-risk supraclavicular nodes. At last follow up in 2016, she had no evidence of disease or subsequent malignancies.

Patient 12

A 23-year-old male was diagnosed with a WHO grade II astrocytoma in 2008 leading to his LFS diagnosis caused by a 742C>T mutation. This was treated initially with gross total resection, but the tumor recurred in September of 2011. The recurrent tumor was treated with RT alone and he received 50.4 Gy in 28 fractions completing treatment in December of 2011. In August of 2015, a repeat MRI demonstrated new growth in the prior resection cavity. The growth was resected with pathology showing a WHO grade III astrocytoma and he was started on chemotherapy. Despite treatment consisting of re-resection and reirradiation, the tumor continued to progress and the patient passed away in December of 2016.

Patient 13

A 35-year-old female was diagnosed with a stage I invasive ductal carcinoma of the right breast in 2004 treated with mastectomy and chemotherapy. In January 2006, the breast cancer recurred in the right chest wall with evidence of disease in the right axillary, supraclavicular, and internal mammary nodes. She was treated with chemotherapy and radiation therapy to 66 Gy in 33 fractions, completing treatment in October 2006. She underwent genetic testing and was diagnosed with LFS due to a 742C>T mutation in the *TP53* gene. At last follow up in October of 2018, she had no evidence of disease or subsequent malignancies.

Patient 14

A 23-year old-male was diagnosed with a stage IIIA colorectal adenocarcinoma in 1992 treated with hemicolectomy and adjuvant chemotherapy. In 2003, he was then diagnosed with a left frontal lobe glioma which was treated with a sub-total resection. In 2005, he had evidence of tumor recurrence and the patient underwent RT receiving 54 Gy in 30 fractions which resulted in a complete remission. He remained disease-free until September of 2010 when a high-grade pleomorphic sarcoma was discovered in his mediastinum. This was treated with surgical resection, chemotherapy, and RT. He had a complete response but the sarcoma recurred in December of 2011. Due to personal and family history of cancer, he underwent genetic testing and was diagnosed with LFS caused by an exon 1 deletion in the *TP53* gene. Despite multiple re-resections and aggressive treatment, his disease continued to progress leading to his death in April of 2013.

Patient 15

A 12-year-old male was diagnosed with a stage IIA high-grade osteosarcoma of the left calf in 2011 leading immediately to the diagnosis of LFS caused by an exon 1 deletion in the TP53 gene. He was treated with amputation and pre- and post-operative chemotherapy. In May of 2013, metastatic disease was discovered in the right lung which was surgically resected. In December of 2013, metastatic disease was discovered in the L1 vertebral body which was treated with palliative RT. He received 16 Gy in 1 fraction in February of 2014 and passed away soon after.

Patient 16

A 29-year-old male with known LFS caused by a 1040C>A mutation in the *TP53* gene was diagnosed with a stage IIB osteosarcoma of his left fifth rib in July of 2015. He was treated with surgical resection and received post-op chemotherapy out of concern for a positive surgical margin. RT was initially avoided due to his known LFS status, however his cancer rapidly progressed. In March of 2016, his large tumor was surgically debulked and he underwent adjuvant/palliative RT. He continued to receive RT up until his death in April 2017.
